# Evaluation of the appropriateness of using glucometers for measuring the blood glucose levels in mice

**DOI:** 10.1038/srep25465

**Published:** 2016-05-06

**Authors:** Yu Togashi, Jun Shirakawa, Tomoko Okuyama, Shunsuke Yamazaki, Mayu Kyohara, Ayumi Miyazawa, Takafumi Suzuki, Mari Hamada, Yasuo Terauchi

**Affiliations:** 1 Department of Endocrinology and Metabolism, Graduate school of Medicine Yokohama-City University, Yokohama 236-0004, Japan

## Abstract

Glucometers are also widely used in diabetes research conducted using animal models. However, the appropriateness of measuring blood glucose levels using glucometers in animal models remains unclear. In this study, we evaluated the consistency between the blood glucose levels measured by 11 models of glucometers and plasma glucose levels measured by a laboratory biochemical test in blood samples collected by retro-orbital sinus puncture or tail-tip amputation. In both blood samples obtained by retro-orbital sinus puncture and those obtained by tail-tip amputation, 10 of the 11 models of glucometers yielded higher glucose values, while 1 yielded lower glucose values, than the plasma glucose values yielded by the laboratory test, the differences being in direct proportion to the plasma glucose values. Most glucometers recorded higher blood glucose levels after glucose loading and lower blood glucose levels after insulin loading in retro-orbital sinus blood as compared to tail vein blood. Our data suggest that the blood glucose levels measured by glucometers in mice tended to be higher than the plasma glucose levels yielded by the biochemical test under the hyperglycemic state, and that differences in the measured levels were observed according to the blood collection method depending on the glycemia status.

Blood glucose is an essential parameter in the study of metabolism and diabetes. Blood glucose levels measured by laboratory biochemical testing are used for the diagnosis of diabetes and for therapeutic monitoring of diabetic patients^1^,^2^. Glucometers are used for strict self-monitoring of blood glucose levels (SMBG) by the patients themselves in intensive diabetes treatment. Experiments in animal models are critical to investigate the significances of the physiological and pathological findings in diabetic patients. Glucometers have been commonly used for measuring the blood glucose levels in animal models, especially rodents, because the measurements can be carried out easily with only a few μ L of blood. Although many models of glucometers are now available for blood glucose measurements in humans, the appropriateness of using glucometers for measuring the blood glucose levels in mice has not yet been fully assessed.

Several blood sampling methods have been recommended for multiple sampling in mice^3^. Tail-tip amputation is commonly applied, because of the simplicity of the technique and rich vascularization of the rodent tail. Retro-orbital blood sampling consistently yields reasonable volumes of blood and is useful for obtaining non-hemolysed plasma, while hemolysis is often observed in blood samples collected by tail-tip amputation^4^. It has been reported that the blood sampling method employed influences some biological parameters, including the plasma concentrations of glucose and blood glucose levels^5^,^6^. Therefore, we conducted this study to compare the impact of the blood sampling method (tail-tip amputation vs. retro-orbital sinus puncture) on the blood glucose values measured by using glucometers.

In the present study, we determined the consistency between the blood glucose levels measured by glucometers and plasma glucose levels measured by a laboratory biochemical test in mice, and evaluated the appropriateness of using blood glucometers for measuring the blood glucose levels in animal studies using mice.

## Results

### Glucometers yielded higher blood glucose levels than the plasma glucose levels measured by the laboratory biochemical test under the hyperglycemic state

**Figure 1:**
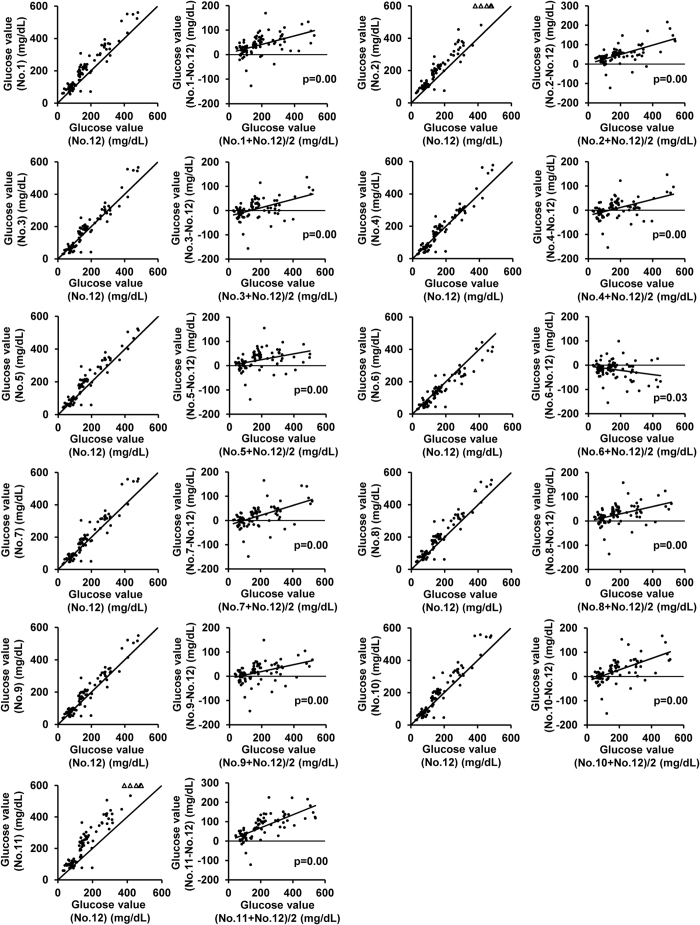
Comparison of the glucose concentrations measured by the glucometers (No. 1–11) and the plasma glucose concentrations measured by a laboratory method in blood samples obtained by retro-orbital puncture. Scatter plot (left) and Bland-Altman plot (right) of measurements by each glucometer (No. 1–11) and plasma glucose levels measured in the laboratory by the mutarotase GOD method (No. 12) in retro-orbital venous blood samples (N =  58). Solid lines in the scatter plots (left) represent lines of equivalence. Solid line in the Bland-Altman plot (right) represents estimated conditional mean values for glucometer coefficients derived by linear regression. Solid circles indicate samples within the measurement limits of the glucometer. Open angles indicate samples showing values over the limit of detection of the glucometers. The p value for the slope is indicated in the Bland-Altman plot.

**Figure 2:**
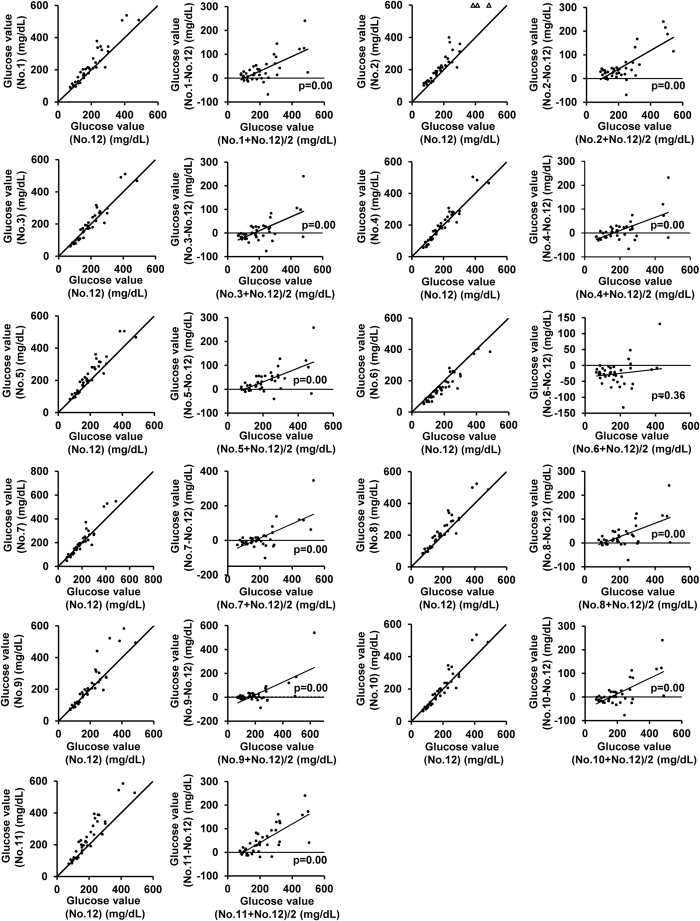
Comparison of the glucose concentrations measured by the glucometers (No. 1–11) and the plasma glucose concentrations measured by a laboratory method in blood samples obtained by tail-tip amputation. Scatter plot (left) and Bland-Altman plot (right) of measurements by each glucometer (No. 1–11) and plasma glucose levels measured in the laboratory by the mutarotase GOD method (No. 12) in tail-tip blood samples (N =  40). Solid lines in the scatter plots (left) represent lines of equivalence. Solid line in the Bland-Altman plot (right) represents estimated conditional mean values for glucometer coefficients derived by linear regression. Solid circles indicate samples within the measurement limits of the glucometer. Open angles indicate samples showing values over the limit of detection of the glucometers. The p value for the slope is indicated in the Bland-Altman plot.

We first compared the blood glucose values obtained with each glucometer model with the plasma glucose levels measured by the laboratory biochemical test in 58 retro-orbital sinus blood samples for 40 blood samples obtained from the tail-tip stump (Figs 1 and 2). Regression lines of measurements by glucometer vs. laboratory biochemical test are also shown in Supplementary Figs S1 and S2. All glucometer models demonstrated higher regression coefficient and lower intercept in tail-tip blood than retro-orbital vessel blood. We next compared blood glucose values of tail-tip blood with plasma glucose concentrations of jugular vein as an independent site and their regression coefficient was slightly smaller (see Supplementary Fig. S3). In both the retro-orbital sinus blood and tail-tip blood samples, all the glucometer models, except model No. 6, recorded consistently higher blood glucose levels than the plasma glucose levels measured by the laboratory test, the differences between the values expanding in proportion to the plasma glucose levels. The average differences between the blood glucose levels measured by the glucometers in the retro-orbital and/or tail-tip blood samples and the plasma glucose levels measured by the laboratory method at plasma glucose levels of < 100 mg/dL, 100–200 mg/dL, 200–300 mg/dL, and > 300 mg/dL are shown in Tables 1 and 2, respectively. Bland-Altman plot analysis revealed significant proportional discrepancies between the measurements by each model of glucometer and the plasma glucose values measured by the laboratory method in the blood samples obtained by retro-orbital sinus puncture (Fig. 1); similar results were obtained for the blood samples obtained by the tail-tip amputation. The proportional discrepancies in the blood glucose values measured by glucometer No. 6 did not reach statistical significance (Fig. 2). Fixed discrepancy, observed regardless of the glucose level, in the positive direction against the laboratory plasma glucose values, was significant in the retro-orbital blood samples measured with glucometer Nos. 1, 2, 5, 7, 8, 9, 10 and 11, and in the tail-tip blood samples measured using glucometer Nos. 1, 2, 5, 8 and 11.

We then investigated the divergence between glucose values by glucometers and plasma glucose levels by the laboratory biochemical method in the oral glucose tolerance test (OGTT) in 18-hour fasted mice or in the insulin tolerance test (ITT) in 2-hour fasted mice. During OGTT, all the glucometers except Nos. 1 and 11 showed lower glucose values than plasma glucose concentration in both retro-orbital samples and tail-tip samples (see Supplementary Fig. S4). The plasma glucose level peaked at 15 min after glucose loading (374.2 ±  22.3 mg/dL), and the percent changes in measurements between glucometers and plasma glucose concentrations were minimized at 15 to 30 min in retro-orbital samples (see Supplementary Fig. S4a,b). Although the plasma glucose concentrations in tail-tip samples also peaked at 15 min (309.9 ±  13.0 mg/dL), the percent changes in glucose values were minimized at 60 min in OGTT (see Supplementary Fig. S4c,d). In ITT, plasma glucose concentrations reached a nadir at 60 min after insulin injection (retro-orbital: 71.8 ±  14.7 mg/dL, tail-tip: 61.9 ±  10.0 mg/dL), and the percent changes in glucose values reached a maximum in both retro-orbital samples and tail-tip samples (see Supplementary Fig. S5).

### Differences in the blood glucose levels measured by the glucometers and the laboratory test between the retro-orbital and tail-tip blood samples

**Figure 3:**
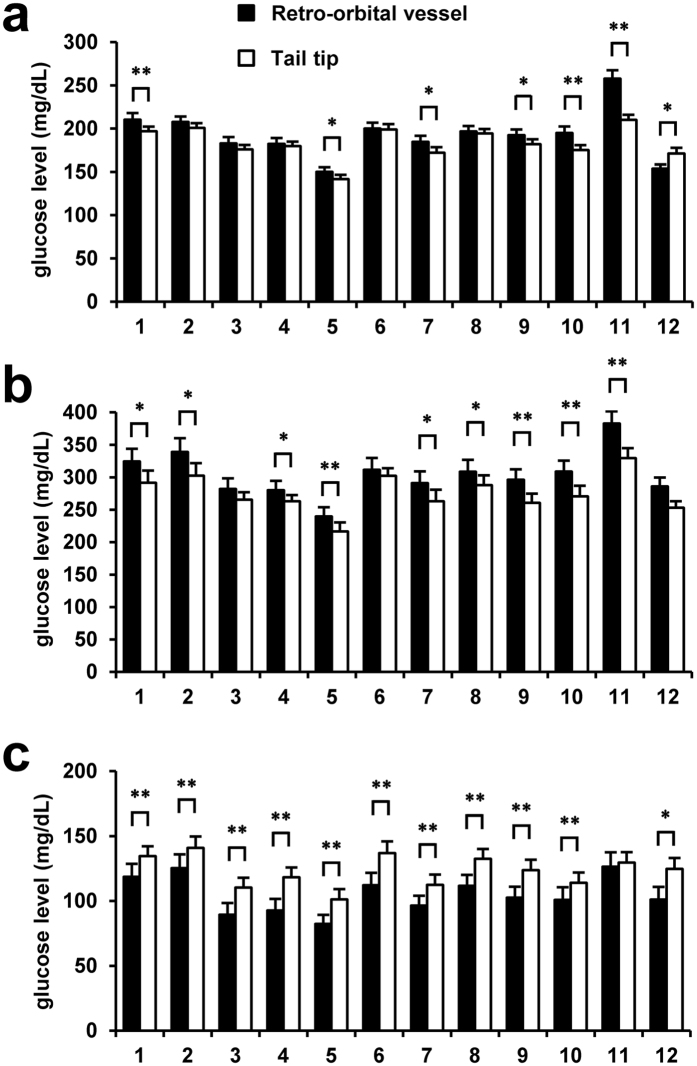
Effect of the method used for the blood collection: retro-orbital vessel puncture vs. tail-tip amputation. Plasma glucose levels were measured in the laboratory by the mutarotase GOD method (No. 12), and blood glucose levels were measured by each glucometer (No. 1–11). (**a**) Sampling in the random-fed status (N =  18). (**b**) Sampling conducted 15 min after oral glucose loading (N =  10). (**c**) Sampling conducted 30 min after intraperitoneal insulin loading (N =  8). *P <  0.05. **P <  0.01. Values are the means ±  SE. Black bars, retro-orbital vessels, White bars, tail-tip.

We next investigated the differences in the blood glucose levels recorded by the glucometers and plasma glucose levels yielded by the laboratory biochemical test between the retro-orbital blood samples and tail-tip blood samples. In the random-fed status, the plasma glucose concentration measured by the laboratory method (No. 12 in Fig. 3a) was significantly lower in retro-orbital sinus blood than in the tail-tip blood (153.7 ±  4.8 mg/dL vs. 171.2 ±  6.6 mg/dL); on the other hand, 5 glucometers showed comparable blood glucose values between the blood samples obtained by retro-orbital sinus puncture and tail-tip amputation, while 6 glucometers showed higher values in retro-orbital blood as compared to tail-tip blood (Fig. 3a). After oral glucose loading, no significant difference in the plasma glucose levels measured by the laboratory method was observed between the retro-orbital and tail-tip blood samples (286.0 ±  13.7 mg/dL vs. 253.1 ±  10.1 mg/dL) (No. 12 in Fig. 3b), whereas the blood glucose levels measured by the glucometers, except for model Nos. 3 and 6, were significantly higher in the retro-orbital blood than in the tail-tip blood (Fig. 3b). After intraperitoneal injection of insulin, the plasma glucose levels measured by the laboratory method were lower in retro-orbital blood than in tail-tip blood (101.1 ±  9.73 mg/dL vs. 124.7 ±  8.4 mg/dL) (No. 12 in Fig. 3c). Contrary to the case in the random-fed status, similar trends to those observed in the values yielded by the laboratory biochemical test were observed in the blood glucose measurements provided by glucometer model Nos. 1–10 (Fig. 3c).

To evaluate the effect of the order of sampling site, we carried out the same examination in reverse order from tail-tip sampling to retro-orbital vessels sampling (see Supplementary Fig. S6). In the random-fed status, the values by the glucometers (see No. 1 to 11 in Supplementary Fig. S6a) and the laboratory method (see No. 12 in Supplementary Fig. S6a) tended to be higher in retro-orbital blood as compared to tail-tip blood. In glucose-loaded mice, no significant difference in the values by both the glucometers (see No. 1 to 11 in Supplementary Fig. S6b) and the laboratory method (see No. 12 in Supplementary Fig. S6b) were observed between the retro-orbital and tail-tip blood samples. After the insulin injection, glucose values by glucometers (see No. 1–11 in Supplementary Fig. S6c) and laboratory biochemical test (see No. 12 in Supplementary Fig. S6c) in retro-orbital blood were higher than in tail-tip blood.

## Discussion

In the present study, we observed varying degrees of discrepancies between the blood glucose levels measured by each of 11 models of glucometers and the plasma glucose level measured by a laboratory biochemical method in mice. It is noteworthy that the blood glucose measurements yielded by the 10 models of glucometers used were higher than the plasma glucose level measured by the mutarotase GOD method, the differences being in direct proportion to the plasma glucose level.

Glucometers are generally calibrated for whole-blood or plasma glucose concentration^7^. Because glucose is dissolved in water component in blood, whole-blood glucose concentration is lower than plasma glucose concentration by 10%. Therefore, the International Federation of Clinical Chemistry and Laboratory Medicine (IFCC) recommended to report plasma glucose concentration^7^. The measurements by all the 11 glucometers in present study were calibrated for plasma glucose concentration, and corrected by hematocrit. The calibration for plasma is less affected by hematocrit than for whole blood in glucose measurements. Furthermore, the calibration for plasma glucose concentration is based on a normal human plasma water concentration (0.93 g/mL). Accordingly, the difference in the distribution of water could cause the measurement discrepancies between glucometers and laboratory biochemical method. In a previous study, measurement using the One Touch II® glucometer (Johnson and Johnson) yielded stepwise increase in the blood glucose levels according to hyperglycemia status in rats with streptozotocin-induced diabetes^8^. These differences was thought to be due, in part, to the higher hematocrit values in rodents, because elevated hematocrit values can cause underestimation of the glucose levels measured by glucometers^8^. In present study, glucometers showed lower glucose values than plasma glucose values during the OGTT in 18-hour fasted mice. This result is thought to be caused by the elevation of the hematocrit after fasting. On the other hand, the impact of the hematocrit on the blood glucose level reportedly differs according to the glucometer model used^9^. The concentration of dissolved oxygen is reported to be another factor influencing the glucose levels measured by the GOD electrode method; hypoxemia and hyperoxemia cause overestimation and underestimation, respectively^10^. Because discrepancies with plasma glucose values have also been observed with methods other than the GOD electrode method, differences in the concentration of dissolved oxygen may explain the discrepancy, at least in part. Reducing substrates, including uric acid and bilirubin are also known to raise the glucose levels measured by glucometers based on the electrode method^11^, while they appear to have no influence on the glucose levels measured by glucometers based on the colorimetric method. Since the discrepancies between the glucose levels measured by glucometers and plasma glucose levels measured in the laboratory were enhanced under the hyperglycemic condition, measuring the blood levels of these intercalators in the hyperglycemic status may be useful to explain the observations.

The AlphaTRAK® (Abbot Laboratory) is a glucometer calibrated for the property of animal blood. The regression line of glucose values obtained by AlphaTRAK® versus plasma glucose values by laboratory biochemical method did not intersect the y-axis at zero, similarly to what is observed with Accu-Chek Advantage® (Roche) glucometer^12^. It is also reported that the direct comparison with the two devices also did not intersect the y-axis at zero^12^. In present study, the regression line of glucose measurements by glucometer and by laboratory biochemical method showed different profiles in respective device. The significant proportional difference was not observed only in the measurements of tail stump blood by No. 6 in this study. In laboratory research using rodents, however, the same device throughout the entire study will provide equal quality of data. Glucometers are useful to screen drugs, to evaluate the efficacy of intervention with diets, exercise, or drugs, and to assess the genetic impact in the preclinical studies. Thus, the reproducibility and consistency of measurements by glucometer might be more important.

Plasma glucose levels measured by the biochemical test in the laboratory were lower in retro-orbital blood than in the tail-tip blood in the random-fed status, while the reverse was observed for the blood glucose levels measured by the glucometers. Blood glucose is delivered to peripheral tissues through arteries, consumed at the capillary level, and returns via the veins. Therefore, blood glucose levels are higher in the order of arteries > capillaries > truncal veins. Changes in the blood glucose levels are dependent on the glucose consumption in the peripheral tissues, which is augmented after glucose or insulin administration. A retro-orbital sample is blood from a vein, and a tail-tip sample is a mixture of blood from the tail artery and tail veins. The difference in the plasma glucose levels in the random-fed condition between the two samples might be explained by this difference in the blood sample composition. The higher blood glucose levels measured by glucometers in retro-orbital blood might be explained by the differences in the concentrations of the intercalators. Glucometers also yielded higher blood glucose values in retro-orbital blood samples as compared to tail-tip blood samples after oral glucose loading, and significantly lower values in retro-orbital blood than in tail-tip blood after insulin administration. These results imply the possibility that retro-orbital blood reflects fluctuations of blood glucose levels earlier than tail-tip blood. In humans, blood glucose measured in the forearm is known to lag more than 30 minutes behind the blood glucose measured at the fingertip during the rapid changes in the blood glucose levels induced by oral glucose loading or intravenous insulin administration^13^. Enhanced distribution of glucose or insulin through blood vessels also possibly contributes to the greater blood glucose fluctuation in the retro-orbital sinus blood than in the tail veins.

Retro-orbital sinus puncture in laboratory rodents is a commonly used technique for blood sampling, although its appropriateness still remains controversial^14^,^15^,^16^. This method may cause distress and injury when implemented inadequately^14^,^17^. A joint EFPIA/ECVAM working group states that this method cannot be recommended, should only be used for animals under anesthesia under compelling circumstances^3^. On the other hand, various methods of anesthesia are reported to have an impact on the blood glucose levels in rodents^5^. Retro-orbital sinus blood from male C57BL/6J mice reportedly showed lower elevations of the plasma corticosterone levels and lesser body weight loss as compared to blood samples obtained by facial vein phlebotomy^16^. However, retro-orbital sinus puncture may exert considerable impact on the animal welfare, by potentially causing subcutaneous hematomas and extensive tissue trauma^16^, which should be considered whenever blood samples are obtained. For measurement of the blood glucose levels, the blood sampling should be minimally invasive and have minimal impact on the stress reaction of the animals, because the release of stress hormones can cause elevation of the blood glucose levels. Furthermore, the order of testing also affected the differences in glucose value between tail-tip sampling and retro-orbital vessels sampling in this study. We also assumed that the blood glucose levels were also elevated due to the stress by fixing the mouse by a retainer during tail-tip blood collection. In fact, continuous blood monitoring using iPro2^®^ (Medtronic) in mice revealed that the process of blood collection by tail-tip amputation without fixing the mouse by a retainer was also associated with a transient increase of the blood glucose levels (Okuyama T, Shirakawa J and Terauchi Y, unpublished data). Therefore, refinement of the blood sampling methods is required.

Investigation of diabetic animal models is essential for elucidation of the pathogenesis of diabetes and development of treatments for this disease. In humans, glycohemoglobin and plasma glucose levels are used as indices for the diagnosis of diabetes, and the diagnostic criteria are based on values associated with the risk of development of microvascular complications^1^,^2^. On the other hand, the diagnostic criteria for diabetes in rodents remain controversial, because it is hard to define the cut-off value for diabetic complication. In the present study, blood glucose levels measured by each model of glucometer showed discrepancies with the plasma glucose levels, the discrepancy increasing in direct proportion to the plasma glucose level; the maximum discrepancy in the plasma glucose range of 200–300 mg/dL was about 30%. In particular, the expanding divergence in the high plasma glucose area can cause overestimation or underestimation under diabetic conditions. Hence, unification of glucose measuring devices, or calibrating the measurements to the plasma glucose value might be required for standardization. Future studies for similar assessment in diabetic mice may be warranted.

In conclusion, we demonstrated the varying profiles provided by 11 glucometers in blood glucose evaluation in mice, and showed discrepancies of the glucose levels between retro-orbital blood samples and tail-tip blood samples after glucose loading or insulin loading. The property of the glucometer and the influence of the method used for blood sampling should be taken into consideration for estimation of the blood glucose levels in mice.

## Methods

### Animals and animal care

Male C57BL/6 J mice were fed a standard-chow diet (MF; Oriental Yeast, Japan) and provided tap water *ad libitum* during the experiments. Blood sampling was performed from 8–13-week-old mice. This study was carried out by experienced personnel at all times, in strict accordance with the recommendations in the Guide for the Care and Use of Laboratory Animals of the Yokohama City University. The protocol was approved by the Yokohama City University Institutional Animal Care and Use Committee (IACUC) (Permit Number: F-A-13-043). All experiments were performed in such a way as to minimize suffering. Animal housing rooms were maintained at a constant room temperature (25 °C) and a 13-h light (8:00 A.M.)/dark (9:00 P.M.) cycle.

### Experimental design

Mice were randomly assigned to the control group (N =  30), glucose administration group (N =  26), and insulin administration group (N =  30). In the control group, blood samples were collected under the random-fed condition. In the glucose administration group, blood samples were collected 15 min after oral loading with glucose (1.5 mg/g body weight) to non-fasted mice. In the insulin administration group, blood samples were obtained 30 min after intraperitoneal injection of human insulin (0.75 mU/g body weight) to non-fasted mice. An oral glucose tolerance test (OGTT) was performed in 18-hour fasted mice by oral administration of glucose (1.5 mg glucose/g body weight). An insulin tolerance test (ITT) was performed in 2-hour fasted mice by intraperitoneally injection with human insulin (0.75 mU/g body weight).

### Blood sampling method

Blood samples were first collected by retro-orbital sinus puncture via the medial canthus of the eye using clean 44.7-μ L heparinized microhematocrit tubes, followed immediately by tail-tip amputation. No anesthesia was used at the time of the blood sampling, so as to avoid unequal variations between animals and also avoid the effects of anesthesia on the blood glucose levels. Mice were fixed by a retainer during blood collection from tail-tip. Blood samples were collected by skilled personnel using the routine technique. The following models of glucometers which were commonly used in clinical practice were used for the measurements: ACCU-CHECK Compact Plus^®^ (Roche Diagnostics, Japan) (No. 1), Medisafe mini^®^ (MS-GR102 TERUMO, Japan) (No. 2), Glutest neo alfa^®^ (Sanwa Kagaku Kenkyusho, Japan) (No. 3), Glutest neo super^®^ (Sanwa Kagaku Kenkyusho, Japan) (No. 4), Glutest mint^®^ (Sanwa Kagaku Kenkyusho, Japan) (No. 5), Freestyle Freedom Lite^®^ (NIPRO, Japan) (No. 6), LIFE CHECK^®^ (eidia, Japan) (No. 7), ACCU-CHECK Aviva Nano^®^ (Roche Diagnostics K K, Japan) (No. 8), Stat Strip Xpress^®^ (Nova biomedical, USA) (No. 9), ONETOUCH Ultra Vue^®^ (Johnson and Johnson, USA) (No. 10), and CareFast C^®^ (NIPRO, Japan) (No. 11). The profile of each glucometer is shown in Table 3. All devices are calibrated for plasma glucose levels. The samples remaining after the glucometer measurements were immediately centrifuged at 3380 *g* for 10 min at room temperature to separate plasma. The plasma glucose levels were determined in the laboratory by LabAssay Glucose (WAKO, Japan), which is shown as No. 12 in Table 3, based on the mutarotase GOD method. The specificity of this laboratory biochemical method is reported to have a margin of error of ±  12% according to the manufacturer’s instruction manual.

### Statistical analysis

Bland-Altman plot analysis was used to compare the blood glucose levels measured by each glucometer (Nos. 1 to 11) with the plasma glucose concentration (No. 12). Values were analyzed using Student’s *T* test to compare the blood glucose levels according to the sampling method.

## Additional Information

**How to cite this article**: Togashi, Y. *et al.* Evaluation of the appropriateness of using glucometers for measuring the blood glucose levels in mice. *Sci. Rep.* **6**, 25465; doi: 10.1038/srep25465 (2016).

## Supplementary Material

Supplementary Information

## Figures and Tables

**Table 1:** The difference between glucometer (No. 1–11) and laboratory method (No. 12) in retro-orbital blood glucose levels.

	Glucose range in No. 12 (mg/dL)							
	0–99		100–199		200–299		300–	
	(mg/dL)	(%)	(mg/dL)	(%)	(mg/dL)	(%)	(mg/dL)	(%)
Glucose value (No. 1–12) (mg/dL)	20.6 ± 3.4	34.7 ± 6.6	43.5 ± 8.8	30.5 ± 5.7	53.8 ± 11.1	20.1 ± 4.0	65.1 ± 13.6	16.5 ± 3.3
Glucose value (No. 2–12) (mg/dL)	30.1 ± 2.6	47.0 ± 5.3	42.9 ± 8.0	30.2 ± 5.2	60.5 ± 14.0	22.9 ± 4.9	101.6 ± 21.9	25.2 ± 5.3
Glucose value (No. 3–12) (mg/dL)	− 9.2 ± 3.3	− 10.3 ± 4.5	10.8 ± 8.3	7.4 ± 5.4	8.5 ± 9.1	3.0 ± 3.3	37.6 ± 19.1	8.5 ± 4.5
Glucose value (No. 4–12) (mg/dL)	− 7.7 ± 3.5	8.1 ± 5.0	10.8 ± 8.3	7.5 ± 5.4	8.3 ± 7.6	2.9 ± 2.8	35.1 ± 20.1	7.7 ± 4.8
Glucose value (No. 5–12) (mg/dL)	6.1 ± 3.2	11.9 ± 5.0	30.7 ± 8.7	21.5 ± 5.6	38.0 ± 9.7	14.2 ± 3.5	26.2 ± 10.9	6.8 ± 2.8
Glucose value (No. 6–12) (mg/dL)	− 8.5 ± 3.9	− 9.5 ± 5.3	− 11.3 ± 7.1	− 7.3 ± 4.5	− 26.6 ± 9.3	− 10.0 ± 3.3	− 52.4 ± 14.2	− 13.6 ± 3.7
Glucose value (No. 7–12) (mg/dL)	− 0.5 ± 3.1	2.3 ± 4.6	16.3 ± 8.9	11.3 ± 5.7	28.3 ± 11.0	10.5 ± 4.1	53.1 ± 19.7	12.8 ± 4.8
Glucose value (No. 8–12) (mg/dL)	13.0 ± 3.4	22.3 ± 5.5	29.0 ± 8.6	20.2 ± 5.6	37.7 ± 9.8	14.4 ± 3.6	48.0 ± 14.1	12.0 ± 3.4
Glucose value (No. 9–12) (mg/dL)	3.6 ± 3.2	8.7 ± 5.0	18.2 ± 8.8	13.1 ± 5.7	22.8 ± 10.2	8.7 ± 3.7	39.3 ± 13.7	9.7 ± 3.4
Glucose value (No. 10–12) (mg/dL)	− 1.1 ± 3.2	8.7 ± 4.6	23.8 ± 9.5	16.2 ± 6.0	42.8 ± 10.8	16.3 ± 3.9	64.2 ± 18.5	16.0 ± 4.7
Glucose value (No. 11–12) (mg/dL)	27.2 ± 3.5	44.1 ± 6.6	72.3 ± 11.3	49.7 ± 7.4	109.6 ± 13.9	41.3 ± 4.8	130.0 ± 14.0	33.5 ± 3.7

values are mean ±  SE. Sample number at − 100 mg/dL, 100–200 mg/dL, 200–300 mg/dL and 300– mg/dL glucose range is 29, 33, 13 and 10 respectively.

**Table 2:** The difference between glucometer (No. 1–11) and laboratory method (No. 12) in tail-tip blood glucose levels.

	Glucose range in No. 12 (mg/dL)							
	–99		100–199		200–299		300–	
	(mg/dL)	(%)	(mg/dL)	(%)	(mg/dL)	(%)	(mg/dL)	(%)
Glucose value (No. 1–12) (mg/dL)	13.1 ± 3.7	16.1 ± 4.3	18.2 ± 4.7	11.9 ± 3.4	39.0 ± 20.1	16.8 ± 8.2	91.1 ± 32.2	25.6 ± 9.7
Glucose value (No. 2–12) (mg/dL)	31.4 ± 3.6	37.3 ± 4.7	23.8 ± 3.6	15.9 ± 2.4	47.4 ± 21.3	20.8 ± 8.8	144.4 ± 40.8	39.1 ± 12.0
Glucose value (No. 3–12) (mg/dL)	− 11.9 ± 2.6	− 13.5 ± 2.4	− 3.2 ± 4.8	− 3.0 ± 3.3	13.5 ± 14.5	6.2 ± 5.9	57.4 ± 36.3	15.9 ± 10.8
Glucose value (No. 4–12) (mg/dL)	− 16.9 ± 5.4	− 19.3 ± 5.4	1.9 ± 3.9	0.3 ± 2.8	13.0 ± 12.4	5.8 ± 5.0	52.7 ± 35.7	14.7 ± 10.6
Glucose value (No. 5–12) (mg/dL)	5.4 ± 4.6	7.9 ± 5.0	20.7 ± 5.2	13.2 ± 3.4	48.4 ± 15.1	20.8 ± 6.4	72.1 ± 31.6	20.8 ± 9.2
Glucose value (No. 6–12) (mg/dL)	− 17.9 ± 4.6	− 20.6 ± 4.0	− 27.7 ± 4.1	− 18.8 ± 2.8	− 29.6 ± 29.6	− 11.9 ± 6.3	− 27.4 ± 27.0	− 7.1 ± 7.8
Glucose value (No. 7–12) (mg/dL)	− 5.7 ± 5.6	− 7.0 ± 6.6	− 6.2 ± 3.6	− 5.1 ± 2.5	15.9 ± 20.6	7.6 ± 8.3	74.7 ± 38.5	19.5 ± 11.7
Glucose value (No. 8–12) (mg/dL)	10.1 ± 4.3	12.5 ± 4.7	15.9 ± 4.5	10.1 ± 3.0	36.5 ± 17.9	15.9 ± 7.3	74.4 ± 35.6	20.8 ± 10.6
Glucose value (No. 9–12) (mg/dL)	− 1.4 ± 3.7	− 1.2 ± 3.8	4.7 ± 4.0	2.7 ± 2.8	11.9 ± 16.3	5.7 ± 6.5	74.7 ± 41.3	19.9 ± 11.9
Glucose value (No. 10–12) (mg/dL)	− 9.1 ± 3.5	− 10.0 ± 3.4	− 1.6 ± 4.1	− 1.9 ± 2.9	20.7 ± 18.7	8.9 ± 7.6	70.4 ± 38.5	19.2 ± 11.5
Glucose value (No. 11–12) (mg/dL)	3.8 ± 4.0	4.9 ± 4.3	26.8 ± 6.9	17.4 ± 4.7	79.4 ± 19.9	33.1 ± 8.2	20.9 ± 73.3	2.8 ± 21.4

values are mean ±  SE. Sample number at − 100 mg/dL, 100–200 mg/dL, 200–300 mg/dL and 300– mg/dL glucose range is 6, 18, 10 and 6 respectively.

**Table 3:** Characteristics of self-monitoring glucometers (No. 1–11) and a laboratory method (No. 12).

No	Product	Measurement principle	Testing time (second)	Sample size (μL)	Sample type	Measurement range (mg/dL)	Calibration
1	ACCU-CHECK Compact Plus^®^	Mut. Q-GDH colorimetric method	5	1.5	Whole blood	10–600	Control solution
2	Medisafe mini^®^	GOD-POD colorimetric method	10	1.2	Whole blood	20–600	Automatic
3	Glutest neo alfa^®^	FAD-GDH electrode method	5.5	0.6	Whole blood	10–600	Automatic
4	Glutest neo super^®^	FAD-GDH electrode method	5.5	0.6	Whole blood	10–600	Automatic
5	Glutest mint^®^	FAD-GDH electrode method	7	0.6	Whole blood Plasma	10–1000 10–600	Control solution
6	Freestyle Freedom Lite^®^	FAD-GDH electrode method	4	0.3	Whole blood	20–500	Control solution
7	LIFE CHECK^®^	GDH electrode method	5	1	Whole blood	20–900	Control solution
8	ACCU-CHECK Aviva Nano^®^	Mut. Q-GDH electrode method	5	0.6	Whole blood	10–600	Code key
9	Stat Strip Xpress^®^	GOD electrode method	6	1.2	Whole blood	20–600	Control solution
10	ONETOUCH Ultra Vue^®^	GOD electrode method	5	1	Whole blood	20–600	Control solution
11	CareFast C^®^	GOD electrode method	5	0.5	Whole blood	20–600	Control solution
12	LabAssay^TM^ Glucose	Mutarotase-GOD method	300	2	Plasma Serum	0–500	
