# Supplementary material for: Evaluation of the appropriateness of using glucometers for measuring the blood glucose levels in mice
Source: Sci Rep. 2016 May 6;6:25465. doi: 10.1038/srep25465 (PMC4858715; doi:10.1038/srep25465)
Supplement: Supplementary Information [file srep25465-s1.pdf]

Supplementary Information:

Supplementary Figures S1-S6

Evaluation of the appropriateness of using glucometers for measuring the blood glucose levels in mice

Yu Togashi, Jun Shirakawa, Tomoko Okuyama, Shunsuke Yamazaki, Mayu Kyohara,

Ayumi Miyazawa, Takafumi Suzuki, Mari Hamada, and Yasuo Terauchi

Supplementary Fig. S1

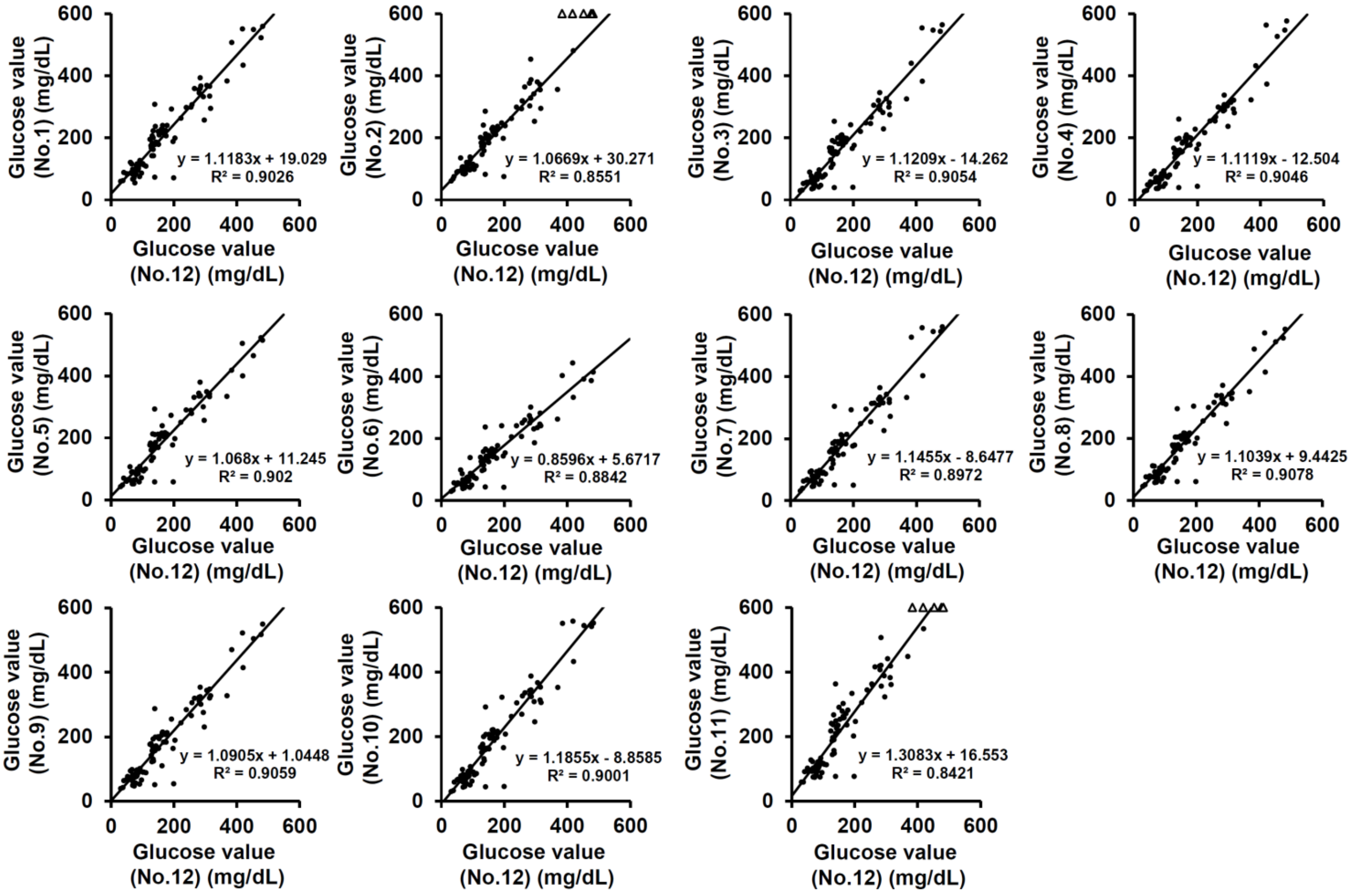

Supplementary Fig. S1. Regression lines of the glucose concentrations measured by the glucometers (No. 1 to 11) vs. the plasma glucose concentrations measured by a laboratory method (No. 12) in blood samples obtained by retro-orbital puncture.

Scatter plot and regression lines of measurements by each glucometer (No.1 to 11) vs. plasma glucose levels measured in the laboratory by the mutarotase GOD method (No.12) in retro-orbital venous blood samples (N = 58). Solid circles indicate samples within the measurement limits of the glucometer. Open angles indicate samples showing values over the limit of detection of the glucometers.

Supplementary Fig. S2

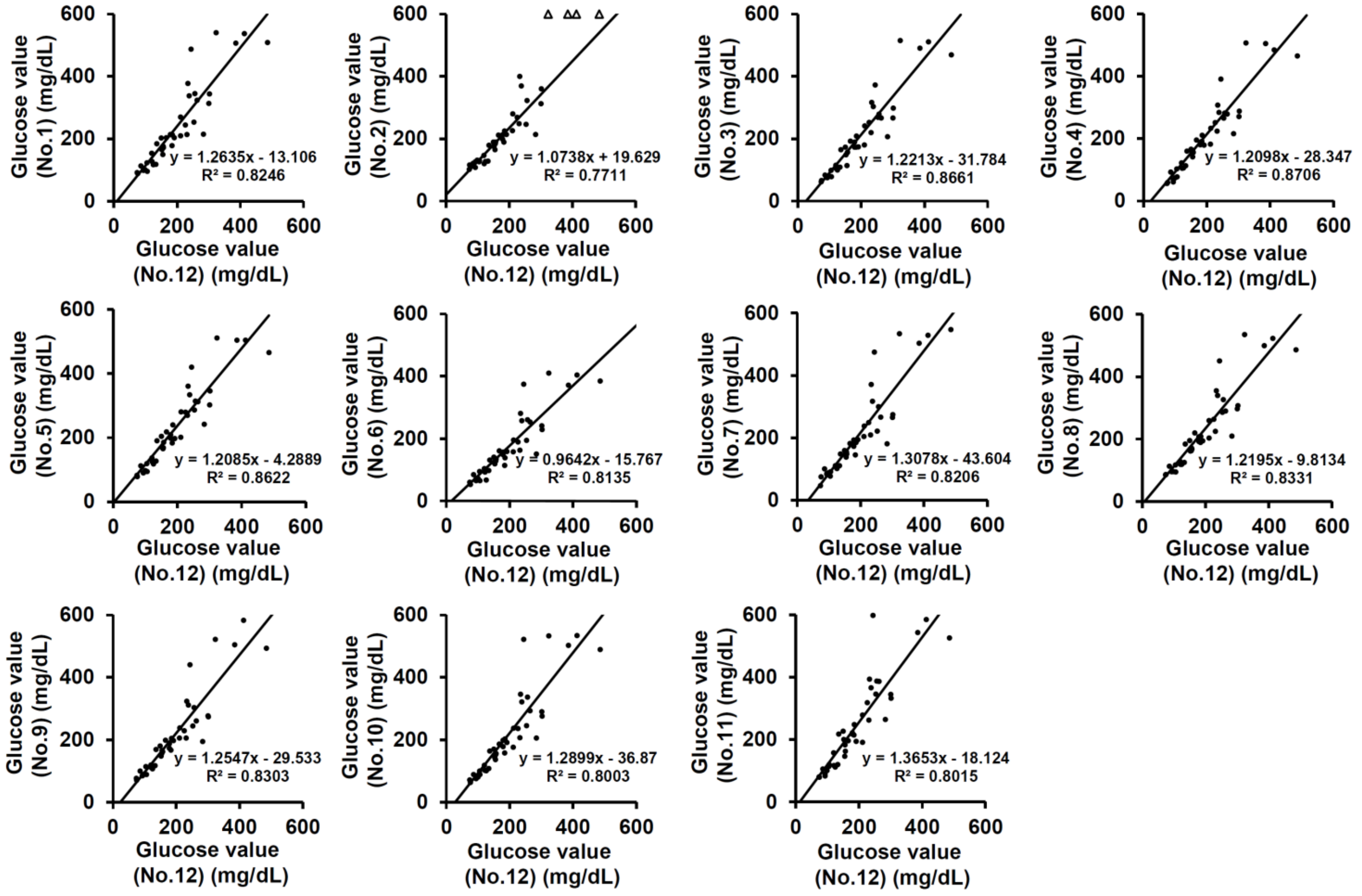

Supplementary Fig. S2. Regression lines of the glucose concentrations measured by the glucometers (No. 1 to 11) vs. the plasma glucose concentrations measured by a laboratory method (No. 12) in blood samples obtained by tail-tip amputation.
Scatter plot and regression lines of measurements by each glucometer (No.1 to 11) vs. plasma glucose levels measured in the laboratory by the mutarotase GOD method (No.12) in tail-tip blood samples (N = 40). Solid circles indicate samples within the measurement limits of the glucometer. Open angles indicate samples showing values over the limit of detection of the glucometers.

Supplementary Fig. S3

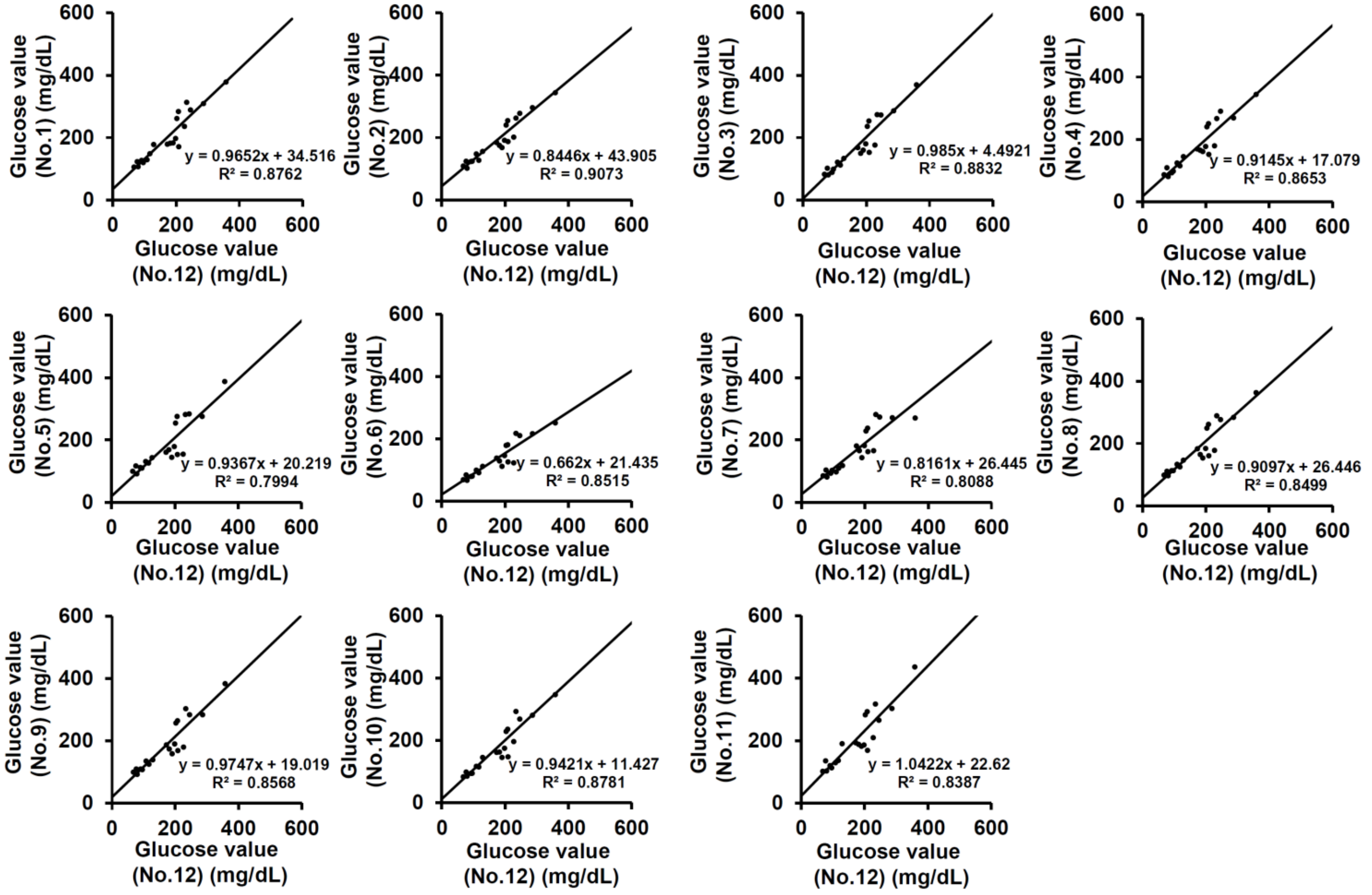

Supplementary Fig. S3. Regression lines of the glucose concentrations in tail-tip blood measured by the glucometers (No. 1-11) vs. the plasma glucose concentrations in jugular-vein samples measured by a laboratory method (No.12).

Scatter plot and regression lines of measurements by each glucometer (No.1 to 11) in tail-tip blood samples vs. plasma glucose levels measured in the laboratory by the mutarotase GOD method (No.12) in jugular-vein blood samples (N = 20).

Supplementary Fig. S4

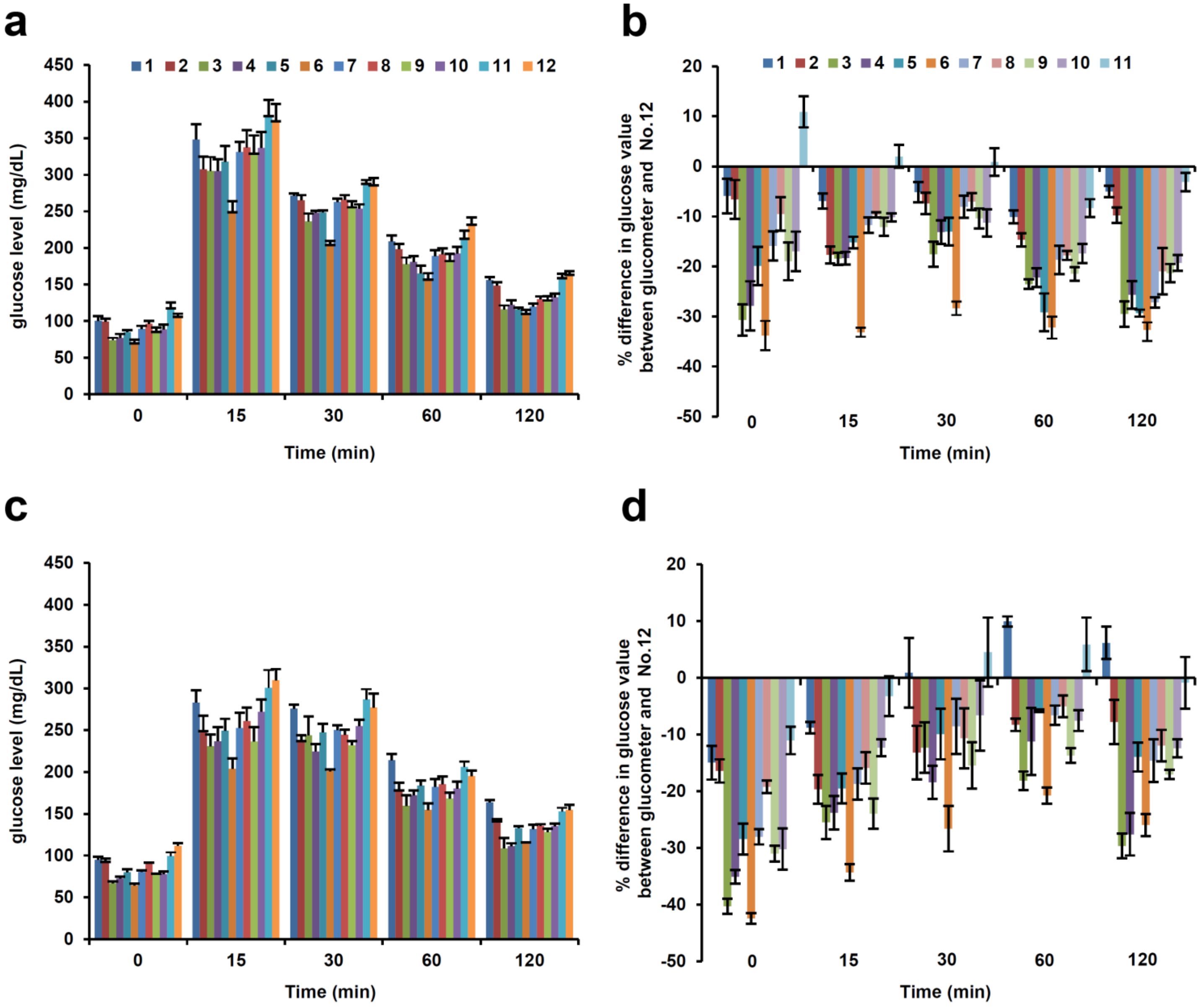

Supplementary Fig. S4. Comparison of the glucose concentrations measured by the glucometers (No. 1 to 11) and the plasma glucose concentrations measured by a laboratory method (No. 12) during OGTT.

Mice were fasted for 18h, and were orally loaded with glucose (1.5 mg/g body weight). Blood samples were collected before (0 min) and 15, 30, 60, 120 min after loading.

Glucose concentrations measured by the glucometers (No. 1 to 11) and the plasma glucose concentrations measured by a laboratory method were compared at each time point.

(a, b) Blood samples by retro-orbital vessel puncture (N=4-5), and (c, d) Blood samples by tail-tip amputation (N=3-5). Values are the means ± SE.

Supplementary Fig. S5

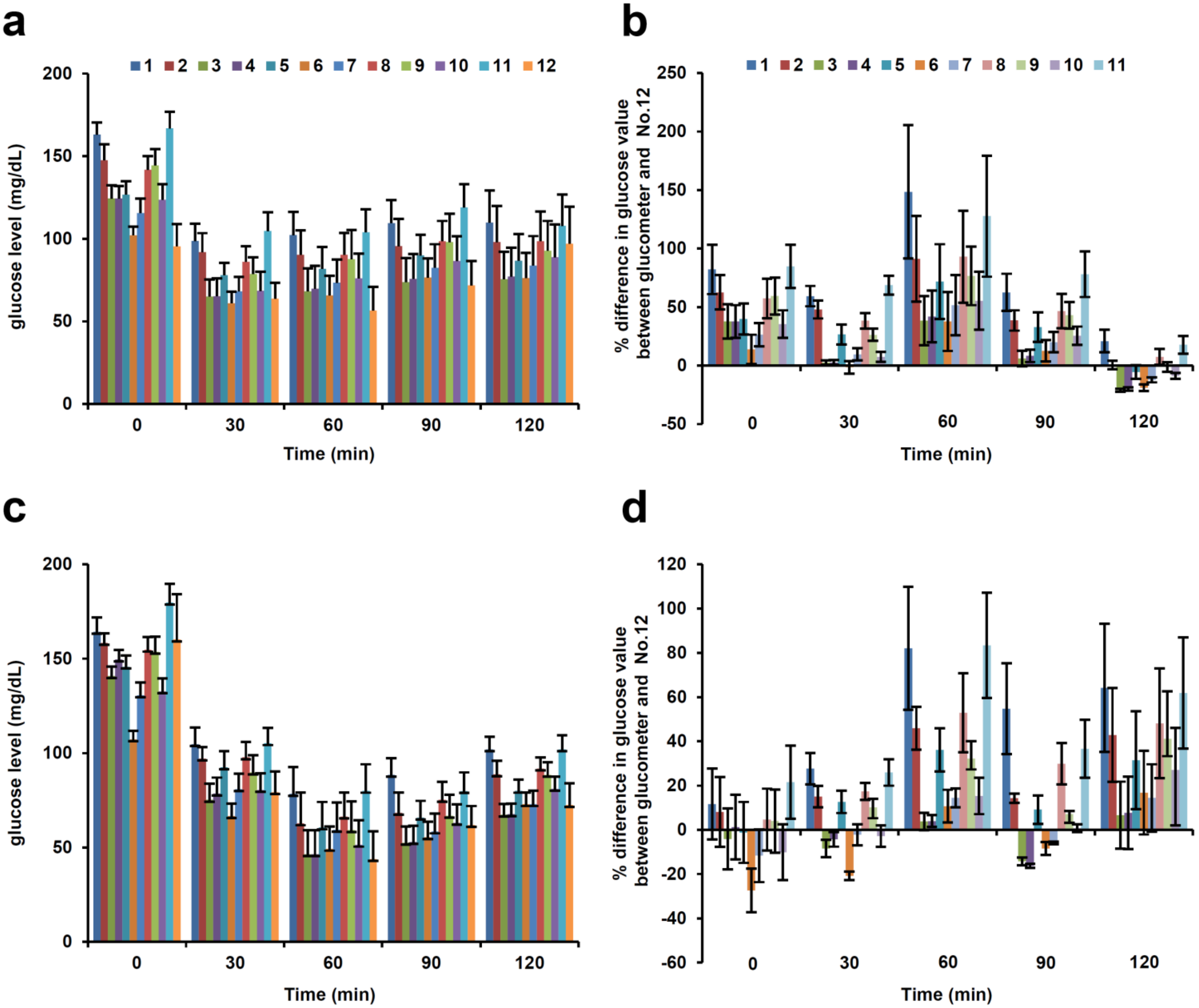

Supplementary Fig. S5. Comparison of the glucose concentrations measured by the glucometers (No. 1 to 11) and the plasma glucose concentrations measured by a laboratory method (No.12) during ITT.

Mice were fasted for 2h, and were intraperitoneally injected with human insulin (0.75 mU/g body weight). Blood samples were collected before (0 min) and 30, 60, 90, 120 min after injection. Glucose concentrations measured by the glucometers (No. 1 to 11) and the plasma glucose concentrations measured by a laboratory method were compared at each time point. (a, b) Blood samples by retro-orbital vessel puncture (N=4-5), and (c, d) Blood samples by tail-tip amputation (N=5). Values are the means ± SE.

Supplementary Fig. S6

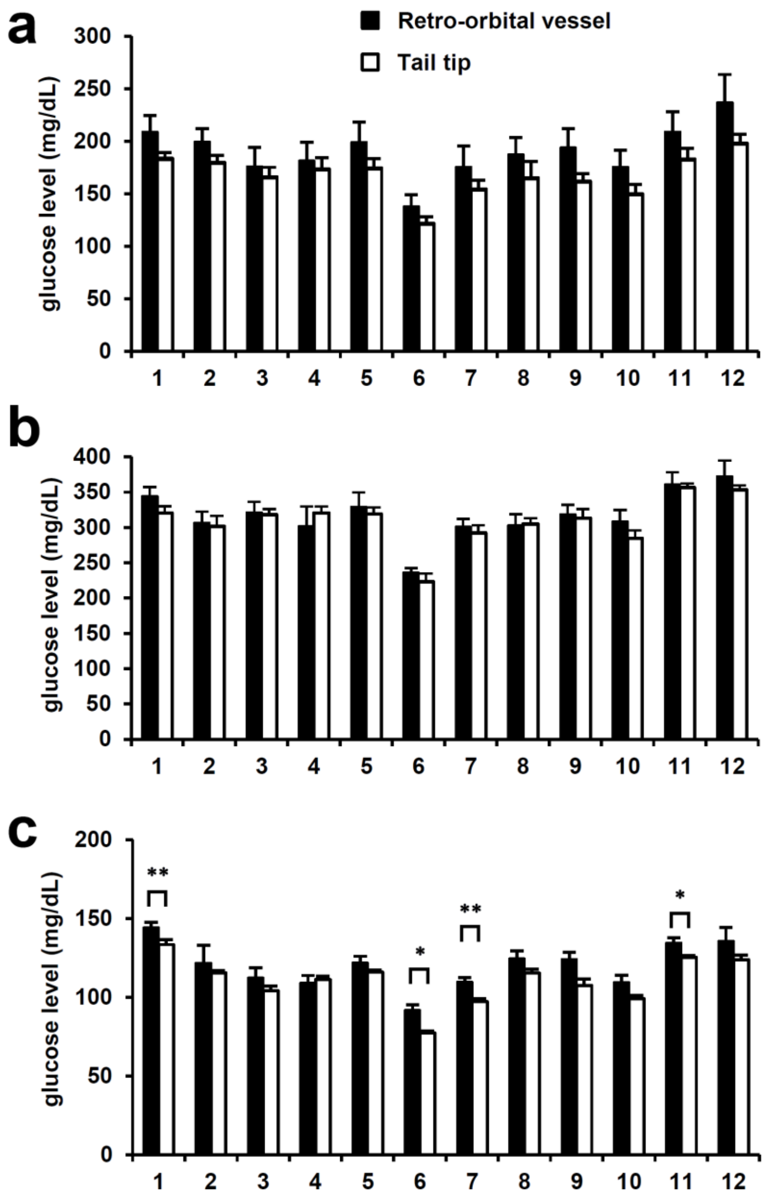

Supplementary Fig. S6. Comparison of the method used for the blood collection: retro-orbital vessel puncture vs. tail-tip amputation, in reverse sampling order.

Blood samples were obtained by tail-tip amputation just followed by retro-orbital vessel puncture. Plasma glucose levels were measured in the laboratory by the mutarotase GOD method (No. 12), and blood glucose levels were measured by each glucometer (No. 1 to 11). (a) Sampling in the random-fed status (N = 5). (b) Sampling conducted 15 min after oral glucose loading (N = 4). (c) Sampling conducted 30 min after intraperitoneal insulin loading (N = 4). *P < 0.05. **P < 0.01. Values are the means ± SE. Black bars, retro-orbital vessels, White bars, tail-tip.
